# Case report: Phenotype expansion and analysis of *TRIO* and *CNKSR2* variations

**DOI:** 10.3389/fneur.2022.948877

**Published:** 2022-08-29

**Authors:** Yuefang Liu, Zhe Liang, Weili Cai, Qixiang Shao, Qiong Pan

**Affiliations:** ^1^Department of Clinical Genetics, Huai'an Maternity and Child Clinical College of Xuzhou Medical University, Huai'an, China; ^2^School of Medical Science and Laboratory Medicine, Jiangsu College of Nursing, Institute of Medical Genetics and Reproductive Immunity, Huai'an, China; ^3^Jiangsu Key Laboratory of Medical Science and Laboratory Medicine, Department of Immunology, School of Medicine, Reproductive Sciences Institute, Jiangsu University, Zhenjiang, China

**Keywords:** *TRIO*, *CNKSR2*, *RAC1*, case report, neurodevelopmental disorders

## Abstract

**Introduction:**

TRIO and CNKSR2 have been demonstrated as the important regulators of RAC1. TRIO is a guanine exchange factor (GEF) and promotes RAC1 activity by accelerating the GDP to GTP exchange. CNKSR2 is a scaffold and adaptor protein and helps to maintain Rac1 GTP/GDP levels at a concentration conducive for dendritic spines formation. Dysregulated RAC1 activity causes synaptic function defects leading to neurodevelopmental disorders (NDDs), which manifest as intellectual disability, learning difficulties, and language disorders.

**Case presentation:**

Here, we reported two cases with *TRIO* variation from one family and three cases with *CNKSR2* variation from another family. The family with *TRIO* variation carries a novel heterozygous frameshift variant c.3506delG (p. Gly1169AlafsTer11), where a prenatal case and an apparently asymptomatic carrier mother with only enlarged left lateral ventricles were firstly reported. On the other hand, the *CNKSR2* family carries a novel hemizygous non-sense variant c.1282C>T (p. Arg428^*^). Concurrently, we identified a novel phenotype never reported in known pathogenic *CNKSR2* variants, that hydrocephalus and widening lateral ventricle in a 6-year-old male of this family. Furthermore, the genotype–phenotype relationship for *TRIO, CNKSR2*, and *RAC1* was explored through a literature review.

**Conclusion:**

The novel variants and unique clinical features of these two pedigrees will help expand our understanding of the genetic and phenotypic profile of *TRIO*- and *CNKSR2*-related diseases.

## Introduction

The neurodevelopmental disorders (NDDs) encompass a wide range of diseases, such as intellectual disability (ID), learning disorders, motor coordination disorders, speech and language disorders, attention deficit hyperactivity disorder (ADHD), and autism spectrum disorder, which are extremely heterogeneous. Overall, the prevalence of NDDs ranges from 9 to 18% (1). Potential pathogenic variants have been identified in more than 1,500 genes involved in different neurodevelopmental processes, such as cell proliferation, neuron migration, synapse formation, and myelination (2). Many NDDs result in a diffuse and variable presentation of neurological symptoms. Phenotype-based cluster analysis established gene–phenotype relationships and revealed compromised molecular processes in specific NDDs subgroups (3). For example, patients with pathogenic variations associated with MAPK pathway typically present short stature, ectodermal anomalies, and ID (4). Genes involved in mitochondrial function are enriched in comorbidities such as epilepsy, metabolic dysfunctions, and myopathy (5). Several human NDDs are known to be caused by variations in Rho family members. One of the most widely studied Rho GTPases is the RAS-related C3 Botulinum Toxin Substrate 1 (RAC1) (6). RAC1 is an important regulator of synaptic function and plasticity by regulating actin polymerization (3). Synaptic function and plasticity play essential roles in cognitive processes, especially in learning and memory formation. Autosomal dominant intellectual developmental disorder-48 (OMIM: 602048) is caused by heterozygous missense variations in the *RAC1* gene. Its clinical features are global developmental delay and moderate to severe ID (7). TRIO and CNKSR2 have been demonstrated as the important regulators of RAC1 (8, 9). TRIO is a guanine exchange factor (GEF) and acts as a key regulator of GTPase RAC1 by accelerating the GDP to GTP exchange. *TRIO* was firstly identified as a candidate gene for ID in 2016 (10). *TRIO*-related intellectual developmental disorders are inherited in an autosomal dominant manner and consistently characterized by global developmental delay, speech impairment, moderate to severe ID and learning disabilities, and macro- or microcephaly. Other phenotypes include behavioral problems and seizures (10–12). CNKSR2 is a scaffold and adaptor protein, which localizes to the dendrites of hippocampal neurons, and helps to maintain Rac1 GTP/GDP levels at a concentration conducive for dendritic spines morphogenesis (9). *CNKSR2* is a causative gene of the Houge type of X-linked syndromic intellectual developmental disorder. Most of the affected men described in the literature exhibited a consistent clinical phenotype of ID, developmental delay, language deficits, and early onset epilepsy (13, 14). However, phenotypic variability complicates the diagnosis of NDDs caused by RAC1-regulating genes.

Here, we report the typical and atypical features of five cases with *TRIO* or *CNKSR2* variations detected *via* whole exome sequencing (WES), respectively. Furthermore, to strengthen our understanding of the phenotype and genotype profile of RAC1-associated NDDs, we performed a literature review of genotype–phenotype analysis for *TRIO, CNKSR2*, and *RAC1*. All these results could facilitate the diagnosis of diseases caused by impaired RAC1 signaling.

## Case presentation

### *TRIO* variation family

**Figure 1:**
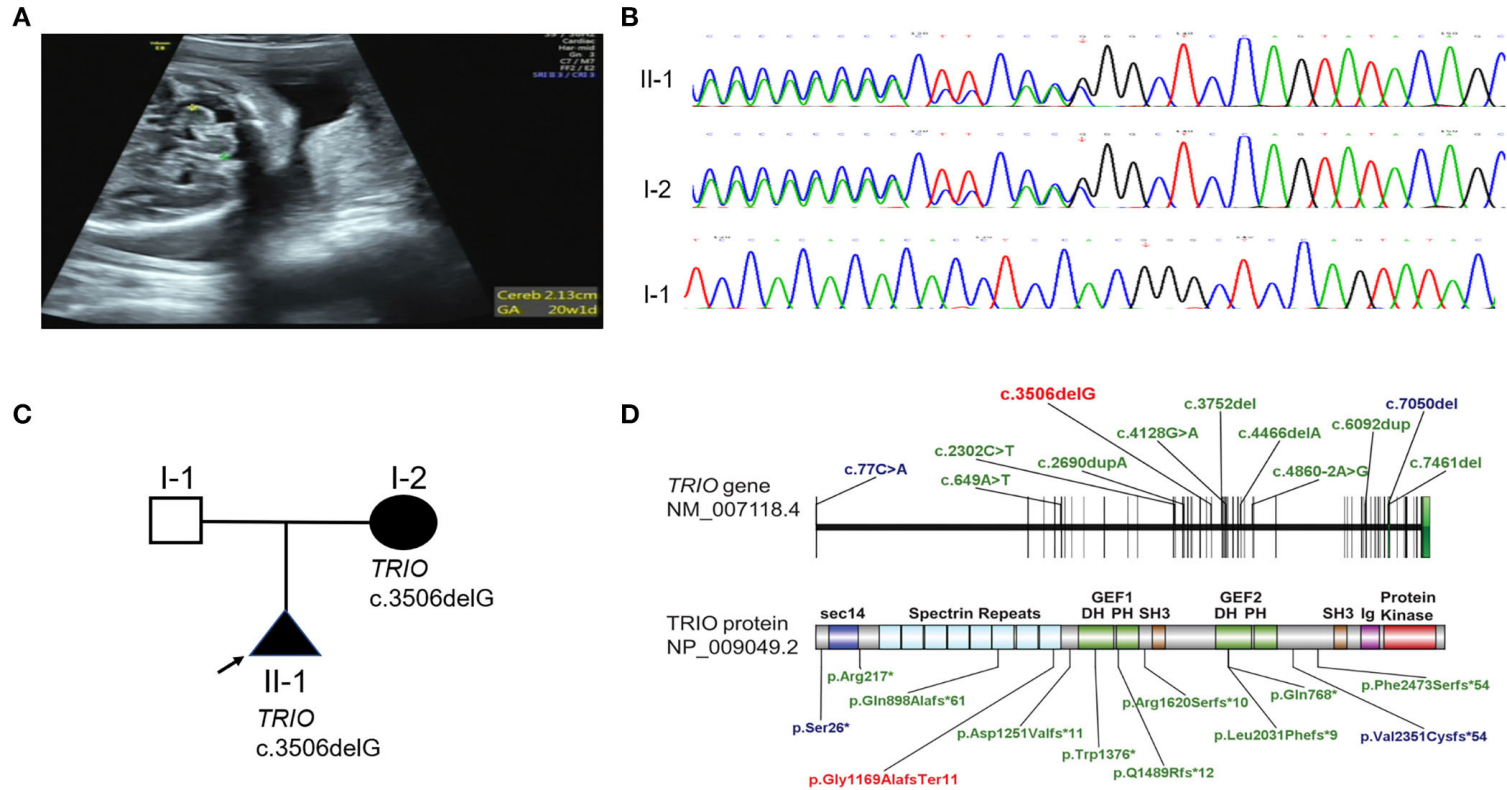
Phenotype and gene variation in *TRIO* cases. **(A)** Delayed development of cerebellar (equivalent to 20 weeks) of case 1 in 25 weeks of pregnancy was detected by prenatal ultrasound. **(B)** *TRIO* c.3506delG variation in case 1 and case 2 were confirmed by Sanger sequencing. **(C)** Family pedigree of *TRIO* variation cases. **(D)** Schematic representation of *TRIO* domains and LOF variations identified in affected individuals (blue: macrocephaly; green: microcephaly; red: variant found by us).

Case 1 was the first pregnancy of non-consanguineous parents. The family history was unremarkable. The mother of this proband at 25 weeks of gestation was referred to our hospital for evaluation of the abnormal fetal ultrasound. Prenatal ultrasound showed a normal head circumference of 22.9 cm. However, the transverse cerebellar diameter was only 2.13 cm (equivalent to 20 weeks) (Figure 1A), and the right and left cerebellar hemisphere diameter was 0.73 and 0.84 cm, respectively (Table 1). In addition, the amniotic fluid index of 10.6 cm suggested polyhydramnios. Brain magnetic resonance imaging (MRI) at 25 weeks showed delayed development of the cerebral cortex such as smooth cerebral surface and a paucity of cingulate sulcus and calcarine sulcus (equivalent to 20 weeks). The transverse diameter of the cerebellum was 2.1 cm and the base of the pons was shallow suggesting delayed development of cerebellum and brainstem (equivalent to 20 weeks). The width of the four ventricles was normal. The pregnancy was terminated at 26 weeks of gestation.

**Table 1:** Clinical phenotype and related gene variations identified in two families with neurological disorders.

**Family**	**Case**	**Age**	**Sex**	**Phenotype**	**Medical imaging**	**Gene**	**Variation**
1	1	25 w	M		The normal head circumference (22.9 cm), shorter transverse cerebellar diameter (2.13 cm), and polyhydramnios (the maximal depth of 10.6 cm) were scanned by prenatal ultrasound in 25 weeks of pregnancy. Delayed development of cerebral cortex (20 weeks), cerebellar and brain stem (21 weeks) were detected by MRI	*TRIO* (NM_007118.2)	c.3506delG (p. Gly1169AlafsTer11)
	2	19 y	F	Normal	A widened left lateral ventricle (1.64 cm) was detected by MRI	*TRIO* (NM_007118.2)	c.3506delG (p. Gly1169AlafsTer11)
2	3	6 y	M	Macrocephaly, delayed motor developmental, autism, cognitive dysfunction, absent language, intellectual disability	A severe hydrocephalus, and a widened bilateral ventricle (2.5 cm) and third ventricle (1 cm) were detected by MRI	*CNKSR2* (NM_014927.3)	c.1282C>T (p. Arg428*)
	4	30 y	F	Normal		*CNKSR2* (NM_014927.3)	c.1282C>T (p. Arg428*)
	5	4 m	F	Delayed development	Upper limit of lateral ventricle (1.0 cm) was detected by prenatal ultrasound at 24 weeks of pregnancy. Abnormally hyperintensity in the left frontotemporal parietal lobe during neonatal stage was detected by MRI	*CNKSR2* (NM_014927.3)	c.1282C>T (p. Arg428*)

w, weeks; y, years; m, months; F, female; M, male; MRI, magnetic resonance imaging.

Skin samples of the fetus and whole blood samples of the parents were collected after obtaining informed consent from the parents. Chromosomal microarray analysis of the fetus indicated a normal karyotype. The whole exomes of proband were captured by using Agilent SureSelectXT Human All Exon V6 (Agilent Technologies, Inc. Santa Clara, CA, USA). High-throughput sequencing was performed by using the Novoseq sequencer from Illumina (Illumina, Inc., San Diego CA, USA). The obtained sequences were aligned to the human genome GRCh37/hg19 reference sequence by BWA (Burrows-Wheeler Aligner) software. A BAM (binary sequence alignment map format) file was produced *via* Picard software. GATK4 (Genome Analysis Toolkit) Realigner Target Creator software and Haplotype Caller software were used to adjust the sequence, extract variants, and generate VCF (Variant Call Format) files. The Annovar software was used to filter and annotate the variant.

A heterozygous frameshift variant c.3506delG (p. Gly1169AlafsTer11) in *TRIO* (NM_007118.2) was screened out *via* WES. No other phenotype-related variants were discovered. *TRIO* is a well-defined haplo-insufficient gene containing 57 exons. Transcripts produced by c.3506delG variant in exon 21 were speculated to undergo non-sense-mediated decay (NMD) resulting in loss of function (LOF) (PVS1) by AutoPVS1 (http://autopvs1.genetics.bgi.com/) (15). So far, this variant was neither found in ExAC nor 1000G (PM2-supporting) and was classified as likely pathogenic according to the ACMG guidelines. The variant was confirmed by Sanger sequencing. The primer sequences for this variant were forward 5′-TAAATGAGGTGCTCGGGGCT-3′ and reverse 5′-ACTCGCTTTCGACTTAGAGG-3′. Sanger sequencing confirmed that the *TRIO* variation was inherited from the asymptomatic mother (Case 2) (Figures 1B,C). The mother of the proband had learning problems and dropped out of school at 12 years old. She demonstrated normal language expression and understanding during counseling. No recognizable facial features were found. To further evaluate the pathogenicity of c.3506delG in *TRIO*, the brain MRI of the mother (case 2) was performed. Only a widened left lateral ventricle (1.64 cm) was indicated (Supplementary Figure 1) (Table 1), and other brain structures were normal (the symmetrical bilateral cerebral hemispheres, the normal gray and white matter, no abnormal focal signal, the normal cerebellum, brainstem, and pituitary gland). LOF variants such as previously reported variants in 14 patients with detailed clinical evaluation and our variant were presented in Figure 1D (blue: macrocephaly; green: microcephaly; red: variant found by us) (10–12, 16).

### *CNKSR2* variation family

**Figure 2:**
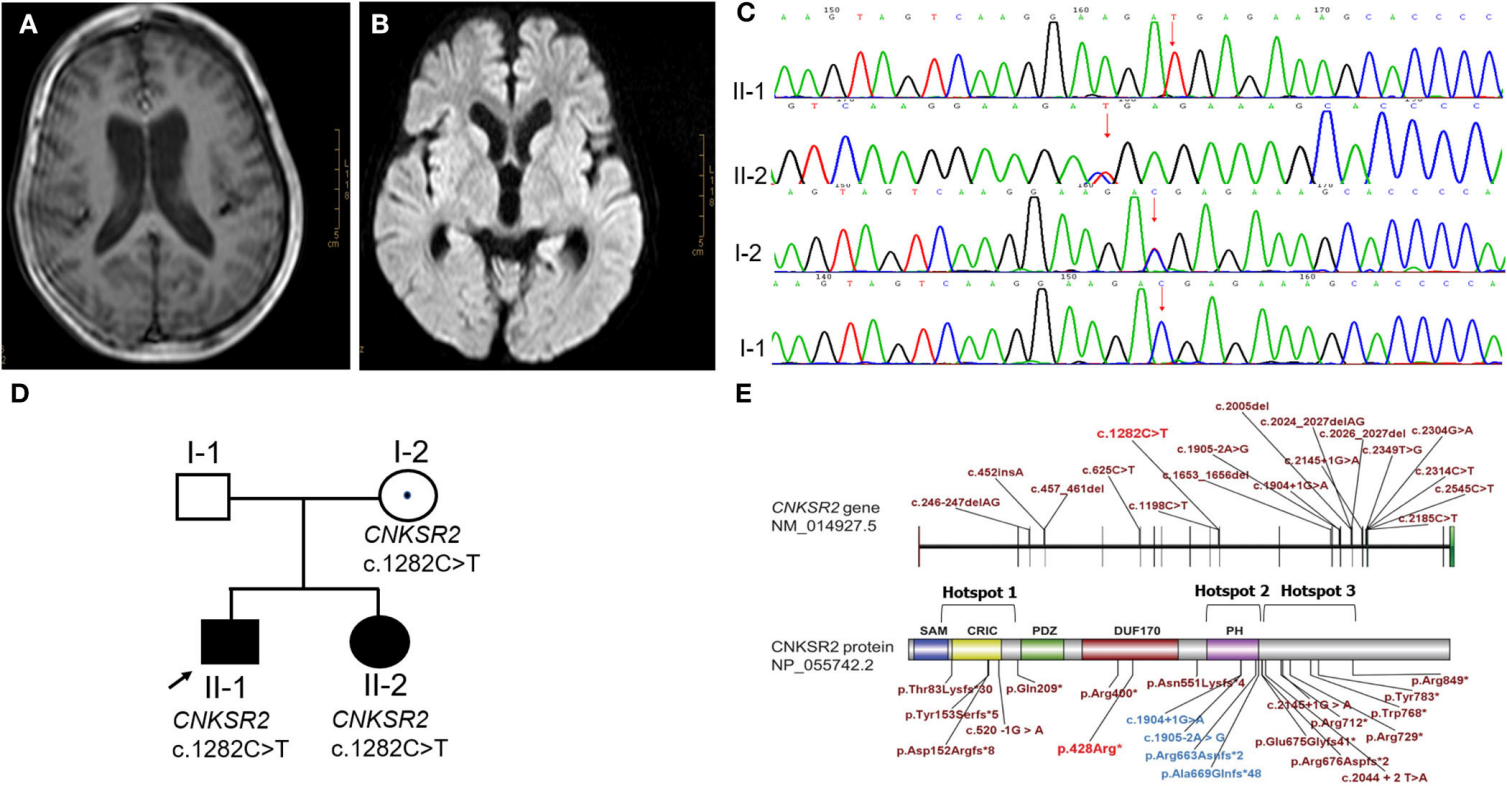
Phenotype and gene variation in *CNKSR2* cases. **(A,B)** A severe hydrocephalus, and a widened bilateral ventricle (2.5 cm) and third ventricle (1 cm) were scanned by MRI. **(C)** *CNKSR2* c.1282C>T variation in *CNKSR2* family was confirmed by Sanger sequencing. **(D)** Family pedigree of *CNKSR2* cases. **(E)** Non-sense variants, splice site mutations, and small insertions/deletions reported in literature (brick red and blue) and this report (red). Hotspot 1: the upstream region of N-terminal PDZ domain; Hotspot 2: the downstream region of C-terminal PH domain; Hotspot 3: PH domain.

A 6-year and 7-month-old boy (case 3) is the first and only child in his family. The family history was also undocumented. The patient was born at term with cesarean section. He walked without support at the age of 18 months. A neuropsychiatric evaluation at 4 years showed obvious symptoms of autism such as poor verbal and non-verbal communication, and cognitive dysfunction. Macrocephaly progressed and brain CT showed a small amount of subdural effusion in both foreheads at 4 years old (Table 1). At 6-year and 7-month-old, his weight was 21.4 kg (−1 SD~median), height was 117 cm (about −1 SD) and head circumference was 54 cm (>2 SD). He could not take care of himself and does not have any expressive speech. The intelligence assessment at the age of 6-year and 7-month-old showed severe ID with an IQ of 40. Brain MRI identified severe hydrocephalus and a widened bilateral ventricle (2.5 cm) and third ventricle (1 cm) (Figures 2A,B). The parents denied any history of epilepsy.

Copy number variation sequencing (CNV-seq) and trio-WES were chosen to screen for causal variants. CNV-seq of proband indicated a normal karyotype. The whole exomes were captured by using BGI V4 chip and sequenced by MGISEQ-2000 (MGI Tech Co. Ltd.). The data were analyzed as described above. A novel hemizygous non-sense variant c.1282C>T (p. Arg428^*^) in the X-linked *CNKSR2* (NM_014927.3) was screened, which was inherited from his unaffected carrier mother. The variants were confirmed by Sanger sequencing. The primer sequences were forward 5′-AAACCTTCCTTGTGAAGACC-3′ and reverse 5′CGCCTAAGTAACTCTCACA-3′ (Figures 2C,D). *CNKSR2* is a well-defined haplo-insufficient gene containing 22 exons. Transcripts produced by c.1282C>T variant in exon 11 was speculated to undergo NMD resulting in LOF (PVS1) by AutoPVS1. The *CNKSR2* c.1282C>T was inherited from the unaffected mother with negative family history and phenotype of proband was unspecific (PS2-moderate). To date, this variant was neither found in ExAC nor 1000G (PM2-supporting) and it was classified as pathogenic according to the ACMG guidelines.

Approximately, 2 years later, this family was admitted to our hospital with the complaint of the widening lateral ventricle of the second pregnancy at 24 weeks (1.00 cm) by prenatal ultrasound (Supplementary Figure 2). Prenatal diagnosis of c.1282C>T in *CNKSR2* was recommended. However, they decided to continue with their pregnancy without the prenatal diagnosis, and subsequently gave birth to a female baby at term with cesarean section. At birth, her weight was 3,500 g. Brain MRI showed abnormally hyperintensity in the left frontotemporal parietal lobe. At 4 months old, physical examination indicated global developmental delay (equivalent to 1 month). Sanger sequencing confirmed the c.1282C>T variant in this second child (Figure 2C).

To date, a total of 28 different variants have been reported in 33 men. Interestingly, all variants were considered as LOF, such as genomic deletions, non-sense variants, splice site mutations, and small insertions or deletions (13, 17) (Figure 2E) (red: non-sense variant found in this report). These results suggest that LOF of *CNKSR2* is the major molecular pathogenic mechanism.

### Genotypes–phenotypes analysis

Although phenotypic variability complicates the diagnosis of NDDs caused by RAC1-regulating genes, there is a significant clinical overlap in *TRIO, CNKSR2*, and *RAC1*. About 30 patients with *TRIO* pathogenic variations, 33 male cases with *CNKSR2* pathogenic variations and seven cases carrying *RAC1* pathogenic variations have been reported in the literature thus far (7, 10–12, 14, 16, 18).

*TRIO* is a multi-domain protein, comprising an N-terminal SEC14 domain, several spectrin repeats, two Rho-guanine exchange factor units (GEFD1 and GEFD2), and a C-terminal serine/threonine kinase domain (16) (Figure 1D). Spectrin repeats domain was known to bind different TRIO regulators to inhibit TRIO-mediated RAC1 activation. While GEFD1 mainly promotes RAC1 activity. Different RAC1 activity resulting from different variations was strongly correlated to the head size and the disease severity (Table 2) (11). *TRIO* variation cases were classified into three groups based on the locations of the variant (11). Group 1 and 2 carry pathogenic missense variants in the hotspot spectrin repeat and GEFD1, respectively. The LOF variants all fall into the third group. Variations in the first group of *TRIO* variations reflecting increased RAC1 activity are associated with severe ID and macrocephaly (Table 2). While group 2 reflecting decreased RAC1 activity showed mild ID and microcephaly (Table 2). Consistently, patients with missense variants in *RAC1* resulting in reduced RAC1 activity showed microcephaly (Table 2) (7). However, the clinical spectrum of group 3 LOF variants in *TRIO* was less well-defined. LOF variants in group 3 were shown in Figure 1D. Remarkably, LOF variants were distributed along the *TRIO* sequence with most cases presenting microcephaly (10, 12, 16) but two carrying c.77C>A (p.Ser26^*^) and c.7050del (p.Val2351Cysfs^*^62) variant with macrocephaly, respectively (11, 12) (Figure 1D, Table 2).

**Table 2:** The comparison of clinical manifestations among *TRIO, CNKSR2*, and *RAC1* variation cases.

**Phenotype**	***TRIO*** **(group 1)**	***TRIO*** **(group 2)**	***TRIO*** **(group 3)**	***CNKSR2*** **(hotspot 1)**	***CNKSR2*** **(hotspot 2)**	***CNKSR2*** **(hotspot 3)**	***RAC1***
ID	9/9 (severe)	7/7 (mild)	14 (ranging from severe to mild)	5/5	9/9	4/4	7/7 (ranging from severe to mild)
Seizures	3/9	0/7	2/5	5/5 (onset age: 2y–3y)	9/9 (onset age: 4d−4y)	3/4 (onset age: 2y)	3/5 (onset age: about 2m−2y)
Learning difficulties	2/9 (moderate) 7/9 (severe)	6/6 (moderate)	14 (ranging from severe to mild)	5/5	9/9	4/4	7/7
Language defect	6/9 (minimal-to-no speech) 3/9 (mild)	2/7 (minimal-to-no speech) 5/7 (mild)	4/12 (minimal-to-no speech) 7/12 (mild)	4/5 (minimal-to-no speech) 1/5 (mild)	6/9 (minimal-to-no speech) 3/9 (mild)	2/4 (minimal-to-no speech) 1/4 (mild) 1/4 (normal)	3/7 (minimal-to-no speech) 2/7 (mild)
OFC	9/9 (macrocephaly)	6/7 (microcephaly)	12/14 (microcephaly) 2/14 (macrocephaly)	Normal	Normal	1/32 (microcephaly)	4/7 (microcephaly) 2/7 (macrocephaly)
ADHD	4/9	4/7	9/13	4/4	9/9	1/4	1/4
Abnormal brain MRI	1/9	0	0	1/5	0	0	6/6

ID, intellectual disability; OFC, occipital frontal circumferences; group 1: missense variations in spectrin repeats domain; group 2: missense variations in GEFD1 domain; group 3: loss of function variations; d, days; m, months; y, years; hotspot 1: the upstream region of N-terminal PDZ domain; hotspot 2: the downstream region of C-terminal PH domain; hotspot 3: PH domain. ADHD, attention deficit hyperactivity disorder.

Two isoforms of CNKSR2, isoform 1 and isoform 3 have been identified in humans and their expressions were only detected in the brain (19, 20). CNKSR2 isoform 1 consists of 1,034 amino acids and contains a sterile alpha motif (SAM) domain, a conserved region in CNKSR (CRIC) domain, a PSD-95/Dlg-A/ZO-1 (PDZ) domain, a pleckstrin homology (PH) domain, and a C-terminal PDZ binding motif (20). CNKSR2 isoform 3 is a truncated form of isoform 1 consisting of 898 amino acids lacking a C-terminal PDZ binding motif (19, 20). No pathogenic LOF variation downstream of the 898th amino acid was reported so far, which may be related with the presence of unaffected isoform 3. Depending on the location, these LOF variants cluster into three hotspots, such as upstream region of the N-terminal PDZ domain (hotspot 1), the downstream region of the C-terminal PH domain (hotspot 2) and the PH domain (hotspot 3, blue) (Figure 2E). Phenotypic differences such as the onset age of seizures, severity of language impairment, and ADHD were not significant between hotspot 1 and 2. However, severe language loss and ADHD were observed at a lower frequency in hotspot 3 (Table 2).

Language impairment was a cardinal feature of *TRIO, CNKSR2*, and *RAC1* variation patients as compared to all other NDDs subgroups, and a high frequency of minimal-to-no speech was observed in *TRIO* group 1 and *CNKSR2* variation cases (Table 2). In addition, structural brain abnormalities in *RAC1* variation cases have frequently been observed, while the seizure is the most common feature in *CNKSR2* variation cases. The onset age of seizures in *CNKSR2* and *RAC1* variation cases was in the first few months or years of life. While in *TRIO* variation cases, the onset age of seizures was later and only reported in *TRIO* groups 1 and 3 (Table 2).

## Discussion and conclusion

In humans, phenotypes caused by *TRIO* variants are variable, even within families with the same variant. They had borderline to severe ID with development delay, learning difficulties, variable speech delay, and abnormal head circumference. The patients also had behavioral problems, such as autistic-like features or ADHD.

Notably, the mother of case 1 presented with learning problems in childhood, normal language expression/comprehension, and enlarged left lateral ventricle in adulthood, who was the first *TRIO* case with incomplete penetrance. However, incomplete penetrance of *TRIO* cases has not been reported previously. The first explanation for incomplete penetrance could be maternal mosaicism. However, we were unable to obtain other tissue samples to confirm this hypothesis. A second explanation is that there may be other genes that interact with *TRIO*, as well as environmental modifiers that we have not considered for this *TRIO* family. Although our analysis did not give an answer to the mechanism of incomplete penetrance in this *TRIO* variation family, the question of incomplete penetrance deserves further study and new hypotheses.

Thus far, all reported cases with pathogenic *TRIO* variants were children and adults. Case 1 in this report was the first prenatal case and head circumference was within the normal range, which has not been reported. Prenatal case 1 was found with delayed development of the cerebral cortex, cerebellum, and brain-stem. Consistently, the prenatal MRI phenotypes such as cerebellar hypoplasia, shortened corpus callosum, and prominent cisterna magna have been described in *RAC1* cases (7), which highlight the role of RAC1 signaling in fetal brain development.

*CNKSR2* variants exhibit delayed development, major ID, speech and language delay, and early onset seizures. Case 3 in our report also showed severe ID, absent language expression, and psychomotor delay. However, he did not exhibit the onset of seizures. All previously reported male patients exhibited the early onset of seizures from neonatal stage to 4 years old with the exception of one Chinese 6-year-old boy reported by Zhang (21). Abnormal brain structures such as hypoplasia corpus callosum and white matter lesion have been described previously (22, 23). Interestingly, hydrocephalus and widened bilateral ventricle were firstly observed in our *CNKSR2* case. Most of the carrier mothers had normal phenotype or some exhibit with only mild learning disability due to X-inactivation in women. The skewed X inactivation rate could explain the different phenotypes of two female carriers. Depending on the location, these LOF variants cluster into three hotspots districts (Figure 2E, Table 2). Notably, a lower frequency of severe language loss and ADHD was observed in hotspot 3. The PH domain is known to stimulate the MAPK pathway and both isoforms of CNKSR2 are localized synaptically through the PH domain (24, 25). Zhang et al. hypothesized that one possible reason for the relatively mild symptoms observed in hotspot 3 might be that the slanted PH domain of the truncated CNK2 protein has acquired a new function (21). However, only four cases with variants in hotspot 3 have been reported and the underlying mechanism needs further investigation.

Notably, both case 2 with *TRIO* variant and case 3 with *CNKSR2* variant presented enlarged lateral ventricle. The delicate balance of cerebrospinal fluid (CSF) circulation may be disrupted in certain neurological diseases, which are associated with hydrocephalus and enlarged lateral ventricle (26). The actin cytoskeleton has been reported to regulate multi-ciliated ependymal cells that line the ventricular walls and are important for the flow of CSF through ciliary beating (27). Thus, disrupted Rac1 signaling might be associated with the ventricle phenotypes in both families.

In conclusion, our findings expand the spectrum of variants and phenotypes of *TRIO*- and *CNKSR2*-related diseases. Phenotypes associated with gene mutations disrupting RAC1 activity overlap sufficiently, which makes this subgroup of NDDs recognizable.

## Data availability statement

The datasets presented in this article are not readily available because of ethical and privacy restrictions. Requests to access the datasets should be directed to the corresponding authors.

## Ethics statement

The studies involving human participants were reviewed and approved by Jiangsu Huai'an Maternity and Child Health Care Hospital (2021042). Written informed consent to participate in this study was provided by the participants' legal guardian/next of kin. Written informed consent was obtained from the individual(s) and/or minor(s)' legal guardian/next of kin for the publication of any potentially identifiable images or data included in this article.

## Author contributions

YL and ZL collected relevant studies. YL wrote the manuscript. WC and QS previewed and re-edited the manuscript. QP made substantial contributions to the analysis, interpretation of the results of genetic analysis and predicting the pathogenicity for the novel variant. WC, QS, and QP contributed to the final approval of the manuscript. All authors contributed to the diagnosis and treatment of the patient. All authors contributed to the article and approved the submitted version.

## Funding

This work was granted by the Maternal and Child Health project of Jiangsu Province (No. F201714 and F201707) and Jiangsu Key Laboratory of New Drug Research and Clinical Pharmacy (XZSYSKF2020024).

## Conflict of interest

The authors declare that the research was conducted in the absence of any commercial or financial relationships that could be construed as a potential conflict of interest.

## Publisher's note

All claims expressed in this article are solely those of the authors and do not necessarily represent those of their affiliated organizations, or those of the publisher, the editors and the reviewers. Any product that may be evaluated in this article, or claim that may be made by its manufacturer, is not guaranteed or endorsed by the publisher.

## Supplementary material

Supplementary Figure 1 A widened left lateral ventricle in case 2 by MRI.

Supplementary Figure 2 A widened lateral ventricle in case 5 by prenatal ultrasound.
