# Supplementary figures and images for: Case report: Phenotype expansion and analysis of TRIO and CNKSR2 variations
Source: Front Neurol. 2022 Aug 29;13:948877. doi: 10.3389/fneur.2022.948877 (PMC9465251; doi:10.3389/fneur.2022.948877)

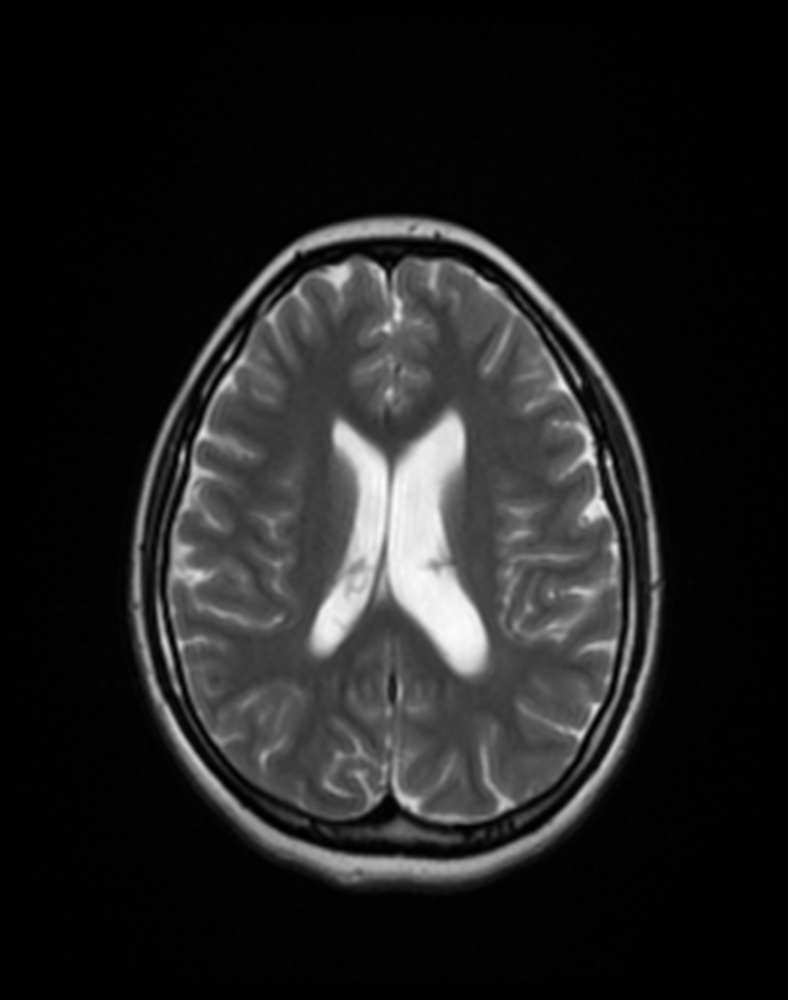

Supplement: Supplementary Figure 1 — A widened left lateral ventricle in case 2 by MRI. [file Image_1.TIF]

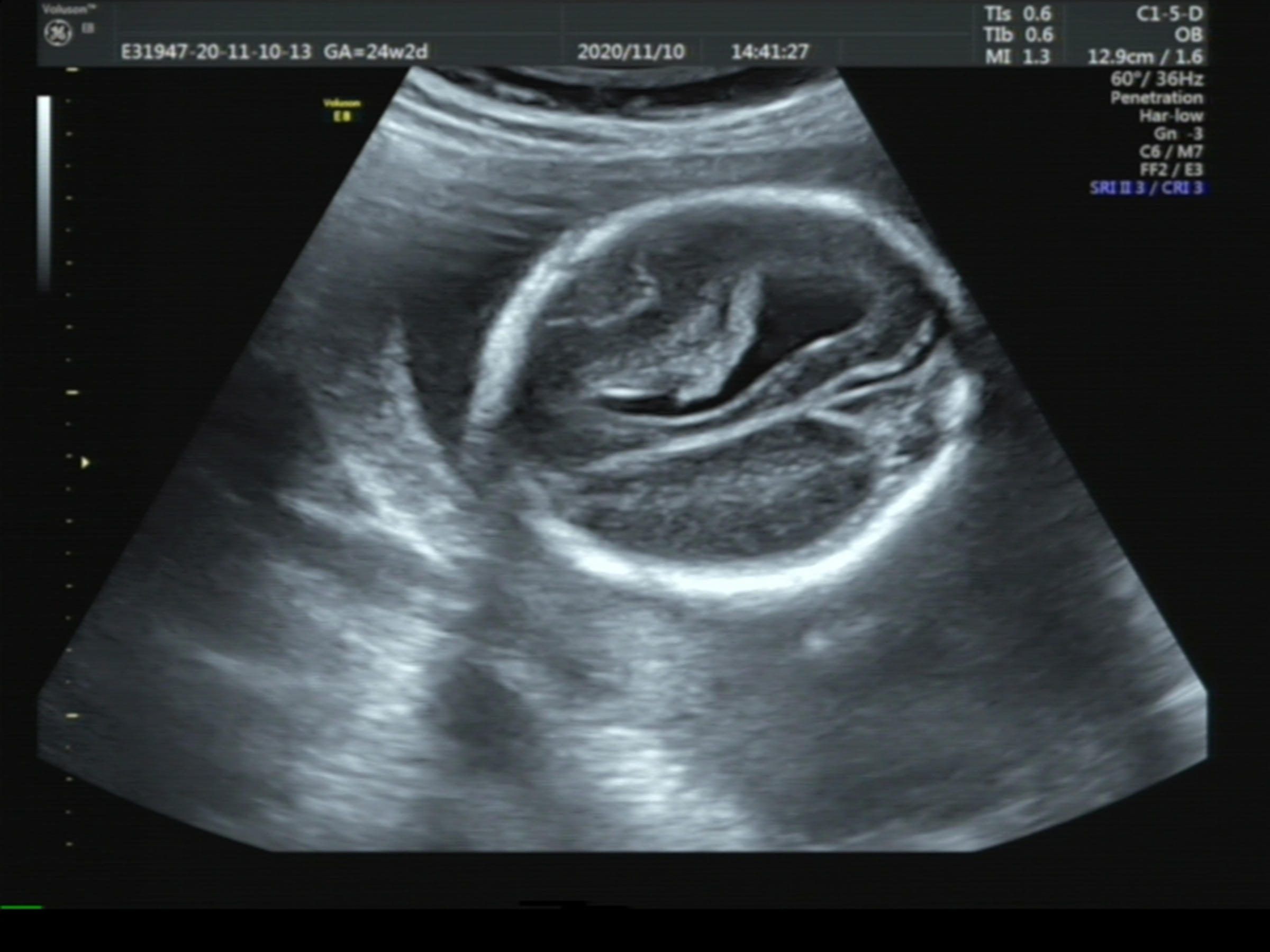

Supplement: Supplementary Figure 2 — A widened lateral ventricle in case 5 by prenatal ultrasound. [file Image_2.TIF]
